# Point-of-care ultrasound to evaluate volume status in congestive heart failure

**DOI:** 10.1186/s44348-026-00078-5

**Published:** 2026-07-07

**Authors:** Nathan C. Shaul, Alan M. Smeltz, Evan J. Raff, Shawn Jia, Lauriane Guichard, Daniel J. Rosenkrans, Jay W. Schoenherr, Jacob D. Acton, Duncan J. McLean, Mark E. Henry, Alexander Doyal

**Affiliations:** 1 Department of Medicine, University of Michigan, Ann Arbor, MI USA; 2 Department of Anesthesiology, University of North Carolina, Chapel Hill, NC USA; 3 Department of Medicine, University of North Carolina, Chapel Hill, NC USA

**Keywords:** Diagnostic ultrasound, Cardiac edema, Congestive heart failure, Central venous pressure, Cardiogenic shock

## Abstract

An accurate volume status assessment is critical to the diagnosis and management of congestive heart failure. Clinically, volume status is typically assessed using a combination of physical examination findings, laboratory values, imaging studies, and invasive procedures. However, these techniques may be unreliable, cost-prohibitive, or impractical. Point-of-care ultrasound (POCUS) has the potential to improve the accuracy of volume assessments with little additional cost to the patient. This review summarizes POCUS techniques that can be used to determine volume status in patients with congestive heart failure.

**Supplementary Information:**

The online version contains supplementary material available at 10.1186/s44348-026-00078-5.

## Introduction

Congestive heart failure (CHF) is a clinical condition in which impaired cardiac function results in elevated filling pressures and decreased cardiac output, triggering a neurohormonal cascade leading to fluid retention [1]. The resultant intravascular hypervolemic state can lead to hydrostatic pulmonary edema due to elevated left atrial pressure, and systemic vascular congestion due to elevated right atrial pressure [1, 2]. Pharmacological diuresis is an essential therapy for volume overload from CHF, and it is critical to accurately assess a patient’s clinical volume status to guide diuretic therapy and other volume-related decisions [3, 4].

Clinical indicators of volume overload include symptoms of dyspnea, weight gain, fatigue, orthopnea, swelling, and paroxysmal nocturnal dyspnea [5, 6]. Physical exam findings may include hypoxia, pulmonary rales, dependent peripheral edema, jugular venous distension, and hepatojugular reflux. However, these findings are poorly sensitive and variably specific for volume overload secondary to CHF (Table 1) [5, 7–14]. Despite their limited diagnostic accuracy, clinical signs and symptoms remain an important component of volume assessment and serial monitoring in decompensated heart failure. Although outpatient telemonitoring programs to assess a patient’s daily weight and symptom burden have been developed, these have failed to show improvements in hospitalization or mortality rates [15].

**Table 1:** Sensitivity and specificity of symptoms, physical exam signs, and noninvasive studies used to diagnose decompensated heart failure

Metric	Sensitivity	Specificity	Reference
Symptom			
Dyspnea	Variable (50%–90%)	Moderate to high (60%–90%)	[8]
Weight gain	Variable (40%–80%)	Moderate (50%–80%)	[9]
Fatigue	Variable (30%–70%)	Moderate (50%–80%)	[9]
Orthopnea	Moderate (50%–80%)	Moderate to high (60%–90%)	[9]
Paroxysmal nocturnal dyspnea	Variable (40%–70%)	Moderate to high (60%–90%)	[9]
Sign			
Pulmonary rales	Moderate to high (60%–90%)	Moderate (60%–80)	[10]
Peripheral dependent edema	Moderate (50%–70%)	Moderate to high (60%–90%)	[10]
Jugular venous distension	Moderate (50%–70%)	Moderate to high (60%–90%)	[10]
Hepatojugular reflux	Moderate (50%–70%)	Moderate to high (60% to 90%)	[10]
Noninvasive study			
BNP/NT-proBNP	High (80%–90%)	Moderate to high (70%–90%)	[11, 12]
Chest x-ray	Moderate to high (70%–90%)	Moderate (60%–80%)	[11, 13]
Transthoracic echocardiogram	High (80%–95%)	Moderate to high (70%–85%)	[7, 14]

BNP, brain natriuretic peptide; NT-proBNP, N-terminal pro–brain natriuretic peptide

Many other modalities have been used to assess volume status in patients with CHF. Brain natriuretic peptide (BNP) is a serological marker that is highly sensitive for decompensated heart failure [5, 16]. However, it can be inaccurate from a variety of factors such as older age, atrial fibrillation, renal failure, and in patients with increased adipose tissue [5]. BNP may also be inaccurate in advanced CHF and may not reliably predict pulmonary artery (PA) pressures, a surrogate for left-sided filling pressures [17]. Chest radiography detects pulmonary edema with high specificity but poor sensitivity; in the inpatient setting, it can be repeated for limited cost and radiation exposure to monitor changes in the severity of edema [5, 18, 19]. Formal transthoracic echocardiography performed by a sonographer is a noninvasive and highly accurate tool for assessing cardiac congestion [5, 19, 20]. Resource limitations, including increased cost and wait times, may make it an impractical tool for serial monitoring of volume status [21, 22]. Impedance cardiography can estimate cardiac output and stroke volume, and has been investigated for volume status assessment, but is not widely available for this use [15].

Right heart catheterization is the gold standard for detecting elevated cardiac filling pressures in decompensated CHF [5], but it is invasive and not practical for serial monitoring. Central venous pressure (CVP) may also be used as a surrogate for cardiac filling pressures, but it requires central venous access [5]. Continuous intracardiac hemodynamic monitoring in critically ill patients can be performed using a PA catheter. This method measures right ventricular and PA pressures but requires central venous access and intensive care unit admission [23]. Implantable hemodynamic monitors such as CardioMEMS Heart Failure System (Abbott) permit longitudinal PA pressure monitoring and can improve hospitalization and mortality rates, but still require an initial invasive procedure with associated risks [15].

Fortunately, point-of-care ultrasound (POCUS), a relatively novel alternative to traditional methods, may help overcome many of the aforementioned limitations in both initial and serial volume assessments [5, 24–26]. Unlike formal transthoracic echocardiography, POCUS can be performed by the treating clinician at bedside, supporting clinical decisions in real-time. Additionally, POCUS can achieve good approximation of hemodynamic parameters without exposing patients to the risks of an invasive procedure. As a result, POCUS functions as an extension of the physical exam and enables rapid qualitative and limited quantitative evaluation [5, 24–26]. This review summarizes POCUS imaging techniques for volume status assessment of patients with known or suspected CHF.

## Venous assessment of right atrial pressure

Right atrial pressure (RAP) is an important metric for evaluating volume status in CHF, as it is often increased in states of impaired cardiac output and intravascular hypervolemia and decreased in intravascular hypovolemia [7]. The absence of valvular separation between the right atrium (RA) and vena cava makes CVP a surrogate measure of RAP [7]. CVP can be measured invasively, but sonographic assessment of the inferior vena cava (IVC) and internal jugular vein (IJV) are useful for noninvasive estimation of both RAP and CVP [7, 25, 27, 28].

### Inferior vena cava

**Fig. 1:**
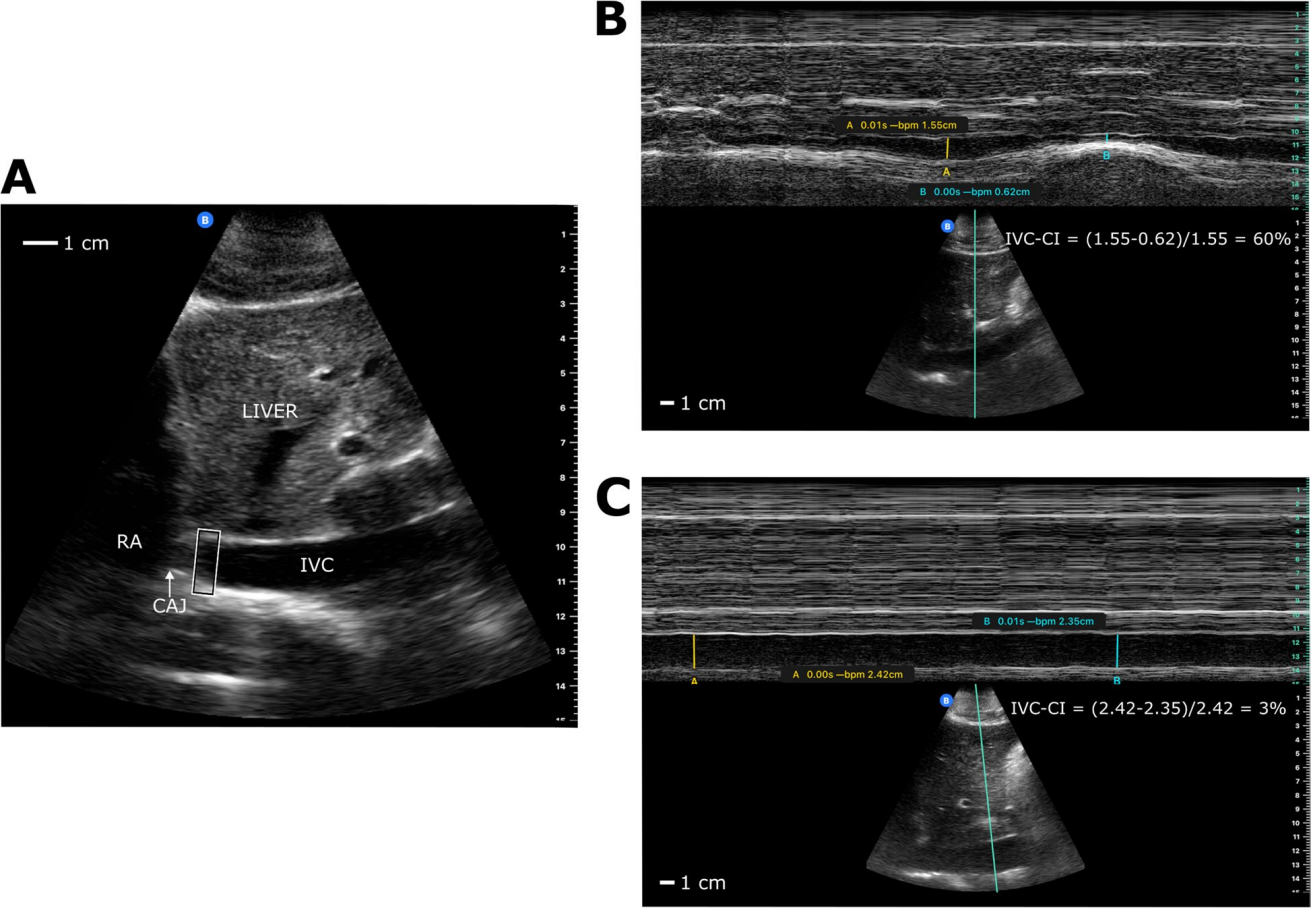
Point-of-care ultrasound (POCUS) images of the inferior vena cava (IVC). **A** A typical POCUS image of the IVC depicting the liver, right atrium (RA), IVC, and cavoatrial junction (CAJ). The box indicates the optimal position for IVC assessment, 1–2 cm from the CAJ. **B** IVC POCUS suggesting the absence of volume overload with an IVC diameter ≤ 2.1 cm and an IVC collapsibility index (IVC-CI) > 50%. (C) IVC POCUS suggesting the presence of volume overload with IVC diameter > 2.1 cm and IVC-CI < 50%

The IVC is visualized using a low-frequency transducer (curvilinear or phased array) in the subcostal position [7, 25, 27]. Sagittal plane imaging produces a longitudinal view of the IVC, which can be traced cephalad to the inferior cavo-atrial junction, where it joins the RA (Fig. 1A, Video S1) [7, 25, 27]. Measurements of IVC diameter throughout the respiratory cycle during spontaneous ventilation, typically taken using M-mode within 1 to 2 cm of the cavoatrial junction, identify the maximum (expiratory) diameter and minimum (inspiratory) diameter [7, 25, 27]. The IVC reaches maximum diameter at end-expiration, and IVC images should accordingly be standardized to phase of the respiratory cycle. The IVC collapsibility index (IVC-CI) is computed by dividing the difference between these diameters by the maximum diameter (Fig. 1B, C) [7, 25, 27]. In a study of 89 spontaneously breathing emergency department patients with symptoms of CHF, an IVC-CI < 30% predicted a clinical diagnosis of acute heart failure with a sensitivity of 80% (95% confidence interval [CI], 63%–91%) and a specificity of 81% (95% CI, 68%–90%) [29].

The IVC maximum diameter and IVC-CI can be used in combination to estimate RAP. A study of 102 patients undergoing right heart catheterization reported that IVC maximum diameter > 2 cm predicted RAP > 10 mmHg with 73% sensitivity and 85% specificity, and IVC-CI < 40% predicted RAP > 10 mmHg with 73% sensitivity and 84% specificity [30]. The European Society of Echocardiography and the American Society of Echocardiography released guidelines for quantification of RAP based on IVC diameter and collapsibility with a sniff test using a diameter threshold of 2.1 cm and a collapsibility threshold of 50% [7, 27, 31]. A maximum IVC diameter ≤ 2.1 cm and IVC-CI > 50% suggests a RAP 0–5 mmHg, whereas diameter > 2.1 cm and collapsibility < 50% reflects a RAP 10–20 mmHg [7, 27, 31]. If neither combination is present, the RAP can be estimated at 8 mmHg [7]. Based on data from 102 patients undergoing right heart catheterization, these criteria have a positive predictive value (PPV) of 89% for RAP 0–5 mmHg, and a PPV of 86% for RAP 10–20 mmHg [30].

IVC ultrasound is useful for estimating RAP and volume overload in acute exacerbations of CHF, but new data suggest that IVC POCUS can facilitate serial monitoring and diuretic titration in the outpatient setting [32, 33]. Advanced practice providers trained in POCUS can assess RAP and achieve measurements consistent with transthoracic echocardiography [32]. Additionally, in a study of 119 patients on outpatient CHF therapy, ultrasound-guided volume assessment predicted changes in diuretic dosing better than patient symptoms, physical exam, and laboratory findings, which the authors attribute to higher interobserver reliability of POCUS [33]; this may, however, simply reflect clinician preference for sonographic data. It is also difficult to subjectively distinguish the net impact of aggregate data points on clinical decision-making from the effect of individual datum. In other words, perhaps POCUS findings consistent with other clinical data reinforce management decisions rather than drive them in isolation.

There is also growing evidence that IVC POCUS can predict heart failure outcomes. Larger median inspiratory IVC diameter measured 1–2 cm from the RA is associated with hospital admission in patients presenting to the emergency department (median, 1.8 cm discharged vs. 2.08 cm admitted; P = 0.014) [34], as well as hospital readmission in patients who are discharged following treatment for CHF [35, 36].

Notably, a wide IVC with limited collapsibility is not specific to volume overload in the setting of heart failure. The list of possible etiologies for these findings is broad, and includes venous pooling after vasovagal syncope, tricuspid regurgitation, sepsis, vasodilatory medications, cardiac tamponade, pulmonary embolism, pulmonary hypertension, and increased intra-abdominal pressure [7]. Other common pitfalls of IVC POCUS include misidentification of bowel or aorta as IVC and underestimation of the IVC diameter by imaging a cross-section that is not in the center of the vessel (Table 2) [26]. However, an IVC assessment by POCUS can yield helpful information to guide diagnosis and management of patients with suspected or confirmed volume overload in the setting of CHF. Importantly, thresholds used to assess maximum IVC diameter and IVC-CI vary among studies. We propose the values of 2.1 cm for maximum IVC diameter and 50% for IVC-CI put forth by the European Society of Echocardiography and American Society of Echocardiography, as these are the most widely validated thresholds for IVC POCUS.

**Table 2:** Ten common pitfalls that occur when using point-of-care ultrasound to assess volume status, as well as techniques that can be used to avoid these pitfalls

No	Pitfall	How to avoid
1	Aorta mistaken for IVC	Trace IVC into RA, view hepatic vein entering IVC
2	IVC sampled > 2 cm from RA	Measure IVC 1–2 cm from cavoatrial junction
3	IVC/IJV interpreted as volume overload with positive pressure ventilation or high PEEP	Interpret IJV/IVC with caution in positive pressure ventilation or high PEEP, consider VExUS
4	IVC interpreted as volume overload in cirrhosis	Interpret IVC assessment with caution in patients with cirrhosis
5	IJV misaligned in long-axis view	Compare to short-axis view, small adjustments to optimize image
6	Hyperdynamic LV interpreted as hypovolemia in low afterload state	Interpret volume status cautiously in low (sepsis) or high (severe HTN/AS) afterload states
7	TR velocity used to estimate PASP in severe TR	Avoid estimating PASP based on TR velocity in severe TR
8	LVOT VTI measured from single beat in atrial fibrillation	Average over > 5 beats to account for beat-to-beat variability
9	B-lines attributed to volume overload in patients with ARDS or fibrosis	Consider clinical context and possible alternatives to pulmonary edema
10	Unilateral B-lines attributed to volume overload	Consider alternatives such as atelectasis, consolidation

ARDS, acute respiratory distress syndrome; AS, aortic stenosis; HTN, hypertension; IJV, internal jugular vein; IVC, inferior vena cava; LV, left ventricle; LVOT, left ventricular outflow tract; VTI, velocity time integral; PASP, pulmonary artery systolic pressure; PEEP, positive end-expiratory pressure; RA, right atrium**;** TR, tricuspid regurgitation; VExUS, Venous Excess Ultrasound Score

### Internal jugular vein

Jugular venous distension (JVD) is commonly assessed on physical examination to estimate RA pressure. The vertical height of distended IJV relative to the sternal angle, or JVD, is added to an assumed right atrial depth of 5 cm to estimate RA pressure in centimeters of water (cm H_2_O) [37]. Previous studies of the diagnostic accuracy of JVD for elevated RAP vary widely, with reported sensitivity 47%–92% and specificity 29%–96% [37–39]. Additionally, adipose tissue may obscure the jugular veins and prohibit accurate assessment of JVD in obese patients [25].

**Fig. 2:**
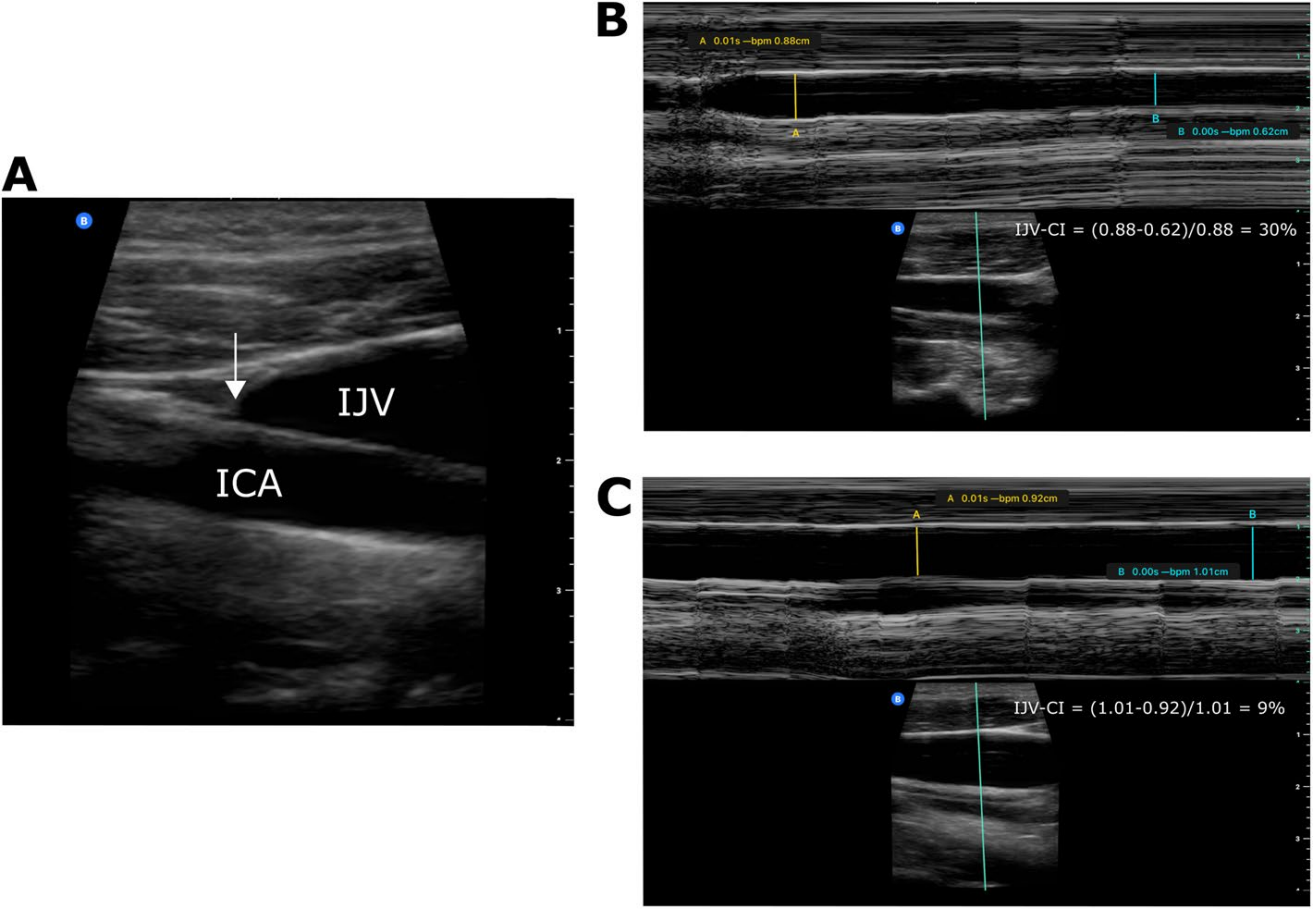
Point-of-care ultrasound (POCUS) of the internal jugular vein (IJV). **A** A typical POCUS image depicting the IJV, internal carotid artery (ICA), and IJV collapse point (arrow). **B** IJV POCUS suggesting the absence of volume overload with IJV collapsibility index (IJV-CI) ≥ 30%. **C** IJV POCUS suggesting the presence of volume overload with IJV-CI < 30%

Ultrasound can be used to enhance visualization of the IJV and more accurately assess jugular venous pressure. The proximal jugular vein is identified in transverse view, then traced in longitudinal view until it collapses or tapers (Fig. 2A, Video S2). The vertical distance between this IJV collapse point and the sternal angle represents the JVD and can be added to the right atrial depth to estimate RAP [40, 41]. While this method may be more accurate than visual inspection of the blood column [42], it relies on the often inaccurate assumed right atrial depth of 5 cm [43]. This can be mitigated by using the distance from the chest wall to the depth of the noncoronary aortic valve cusp in the parasternal long-axis view to approximate the depth of the mid-RA [43].

In a study of more than 100 patients presenting to an emergency department with dyspnea, sonographic JVD greater than or equal to 8 cm H_2_O was highly sensitive, but poorly specific, for CHF diagnosed by echocardiogram (sensitivity, 99% [95% CI, 92.2%–100%]; specificity, 59% [95% CI, 40.9%–74.4%]) and likelihood of serum BNP > 500 pg/mL (sensitivity, 100% [95% CI, 92%–100%]; specificity, 43% [95% CI, 31%–56%]) [44, 45]. These findings suggest that sonographic assessment of JVD is useful for ruling out CHF with volume overload when negative.

IJV-CI, also referred to as respiratory variation in diameter, utilizes respiratory variation in IJV diameter to estimate volume status. The IJV-CI is measured by sonographically visualizing the IJV in the long axis and measuring the maximum and minimum anteroposterior diameters throughout the respiratory cycle, then dividing their difference by the maximum IJV diameter (Fig. 2B, C) [25]. Like the IVC, maximum IJV diameter occurs at end-expiration. In patients with volume overload and elevated RAPs, inspiratory collapse of the IJV may be blunted [25]. IJV-CI is highly accurate in states of volume overload due to cirrhosis. One study measuring IJV-CI in 44 patients presenting to an emergency department with decompensated cirrhosis found that an IJV-CI ≤ 24.8% predicted elevated CVP with 100% sensitivity and 97.1% specificity. In the study, this method was superior to IVC-based assessment for a multitude of reasons, including intrahepatic peri-IVC fibrosis and/or increased intra-abdominal pressure from ascites [46].

In addition to the IJV-CI, respiratory variation can be evaluated using the change in IJV cross-sectional area (CSA) measured at the IJV collapse point. A study of 59 patients with dyspnea in an emergency setting showed that a change in IJV-CSA < 50% throughout the respiratory cycle was 70% sensitive and specific for a BNP > 100 pg/mL [47]. The maximum IJV anteroposterior diameter throughout the respiratory cycle can yield insight into a patient’s volume status. When end-expiratory IJV diameter was measured in the transverse view 2 cm above the clavicle, a diameter ≤ 7 mm was 60% sensitive and 88% specific for a low RAP (< 8 mmHg) assessed by invasive CVP measurement [48]. This finding suggests maximum IJV diameter may be useful in ruling out elevated RAP when it falls within normal limits.

The POCUS-JVD study compared IJV POCUS to right heart catheterization in 176 spontaneously breathing patients and developed a framework for estimating RAP. The authors suggest first measuring IJV-CI at the apex of sternal and clavicular heads of the sternocleidomastoid muscle to screen for elevated RAP; an IJV-CI < 30% is 82% sensitive and 71% specific for a RAP > 10 mmHg [49]. They also show that a maximum IJV diameter at or above 1.2 cm increases the specificity for elevated RAP to 88% [49]. The addition of visible JVD to mid-neck or higher on physical exam further increases the specificity for elevated RAP to 97% [49]. Conversely, an IJV-CI ≥ 30% was 71% sensitive and 82% specific for a RAP < 10 mmHg, and the specificity increases to 94% if the IJV is collapsible on a sniff maneuver [49].

Beyond passive respiratory variation in IJV size, provocative maneuvers such as Valsalva and sniff can yield insight into RAP. The effect of these measures can be assessed using change in IJV-CSA measured at rest, then again during the maneuver. A study of 67 patients demonstrated that a < 17% increase in IJV-CSA with Valsalva predicted RA pressure > 12 mmHg on right heart catheterization with 90% sensitivity and 74% specificity [50]. Alternatively, the IJV distensibility ratio, or ratio of the maximal IJV diameter during Valsalva to rest, can predict both RAP and clinical outcomes. A study of 201 patients with CHF undergoing right heart catheterization found an IJV distensibility ratio > 1.6 predicted RAP ≤ 7 mmHg with a PPV of 94%, and IJV distensibility ratio < 1.6 was associated with increased risk of death and advanced heart failure therapy, suggesting a more severe clinical phenotype [51]. Together, these findings suggest IJV-CSA with Valsalva and IJV distensibility ratio are yet another set of tools for ruling out elevated RAP when normal.

There are several caveats to IJV POCUS for volume status assessment (Table 2). Foremost, positive pressure ventilation increases CVP and may decrease the diagnostic utility for this purpose [52]. As in IVC assessment, positive indicators of elevated RAP are not specific for volume overload due to CHF, as other conditions, such as pulmonary embolism, cardiac tamponade, and hepatic cirrhosis, can increase RAP and JVP [53]. A potential result of these caveats, IJV ultrasound has high sensitivity and moderate specificity for hypervolemia; a recent meta-analysis of 13 studies reported a pooled sensitivity of 84% (95% CI, 70%–92%) and specificity of 70% (95% CI, 55%–82%) [54].

Table 3 summarizes POCUS techniques for evaluating RAP and venous congestion [29, 30, 44–51]. Although threshold values for IJV POCUS vary slightly among studies, we propose routinely comparing sonographic JVD to a threshold of 8 cmH_2_O and IVC-CI to a threshold of 30%. Overall, IJV POCUS, especially with IVC assessment, provides a useful tool to augment physical examination and clinical volume assessment in patients with CHF. When normal, IJV POCUS metrics often suggest that volume overload can be ruled out; when abnormal findings are present, this can provide important data to help guide diagnosis and management.

**Table 3:** Summary of POCUS IVC and IJV measurements to estimate right atrial pressure

POCUS study	Finding	Outcome	Sensitivity (%)	Specificity (%)	PPV (%)	Reference
IVC						
Maximum diameter	> 2 cm	RAP > 10 mmHg	73	85	-	[30]
IVC-CI	< 30%	Acute CHF diagnosis	80 (63–91)^a)^	81 (68–90)^a)^	-	[29]
	< 40%	RAP > 10 mmHg	73	84	-	[30]
IVC maximum diameter + IVC-CI	≤ 2.1 cm + > 50%	RAP, 0–5 mmHg	-	-	89	[30]
	> 2.1 cm + < 50%	RAP, 10–20 mmHg	-	-	86	[30]
	All other	RAP, 8 mmHg	-	-	-	[30]
IJV						
Sonographic JVD	≥ 8 cm H_2_O (5.9 mmHg)	CHF by echo	99 (92–100)^a)^	59 (41–74)^a)^	-	[44]
	≥ 8 cm H_2_O (5.9 mmHg)	BNP > 500 pg/mL	100 (92–100)^a)^	43 (31–56)^a)^	-	[45]
IJV-CI	≤ 24.8%	Elevated CVP	100	97.1	-	[46]
	< 30%	RAP > 10 mmHg	82	71	-	[49]
	≥ 30%	RAP < 10 mmHg	71	82	-	[49]
IJV-CI + maximum IJ diameter	< 30% + > 1.2 cm	RAP > 10 mmHg	-	88	-	[49]
IJV-CI + collapsible on sniff	≥ 40% + present	RAP < 10 mmHg	-	94	-	[49]
IJV-CSA	< 50% change	BNP > 100 pg/mL	70	70	-	[47]
IJV end-expiratory diameter	< 7 mm	RAP < 8 mmHg	60	88	-	[48]
IJV-CSA with Valsalva	< 17%	RAP > 12 mmHg	90	74	-	[50]
IJV distensibility ratio	> 1.6	RAP ≤ 7 mmHg	-	-	94	[51]

BNP, brain natriuretic peptide; CHF, congestive heart failure; CVP, central venous pressure; IJ, internal jugular; IJV, internal jugular vein; IJV-CI, internal jugular vein collapsibility index; IJV-CSA, internal jugular vein cross-sectional area; IVC, Inferior Vena Cava; IVC-CI, inferior vena cava collapsibility index; JVD, jugular venous distension; POCUS, point-of-care ultrasound; PPV, positive predictive value; RAP, right atrial pressure

^a^^)^95% Confidence interval

## Focused cardiac ultrasound

In addition to IJV and IVC POCUS to assess right atrial pressure, focused cardiac ultrasound (FOCUS) can provide valuable information regarding a patient’s hemodynamics and volume status. As part of a comprehensive volume status assessment, it is important to consider the different hemodynamic and physiologic parameters assessed by each modality, which are summarized in Table 4.

**Table 4:** Hemodynamic parameters paired with POCUS studies used to assess them

Hemodynamic parameter	RAP	RV pressure/volume	Pulmonary hemodynamic	Extravascular pulmonary fluid	LV function
POCUS/FOCUS assessment	IJV, IVC	D-shaped LV	TR-derived PASP	B-lines	LVEF, LVOT VTI
Pattern 1: • CHF with volume overload	High	Present	High	Present	Normal (HFpEF) Decreased (HFrEF)
Pattern 2: • Group 1, 3–5 pHTN • Cardiac tamponade • Acute pulmonary embolism	High	Present	High	Absent	Normal^a)^
Pattern 3: • Isolated RV failure	High	Present	Normal	Absent	Normal
Pattern 4 • Flash pulmonary edema • Isolated LV failure • ARDS	Normal	Absent	Normal	Present	Normal (HFpEF, ARDS) Decreased (HFrEF)

Common patterns of hemodynamic findings are listed along with representative pathologies that could produce each pattern

ARDS, acute respiratory distress syndrome; CHF, congestive heart failure; FOCUS, focused cardiac ultrasound; HFpEF, heart failure with preserved ejection fraction; HFrEF, heart failure with reduced ejection fraction; IJV, internal jugular vein; IVC, inferior vena cava; LV, left ventricle; LVEF, left ventricular ejection fraction; LVOT, left ventricular outflow tract; PASP, pulmonary artery systolic pressure; pHTN, pulmonary hypertension; POCUS, point-of-care ultrasound; RAP, right atrial pressure; RV, right ventricle; TR, tricuspid regurgitation; VTI, velocity time integral

^a)^LVOT VTI is low in states of reduced cardiac output from cardiac tamponade or a hemodynamically significant pulmonary embolism

**Fig. 3:**
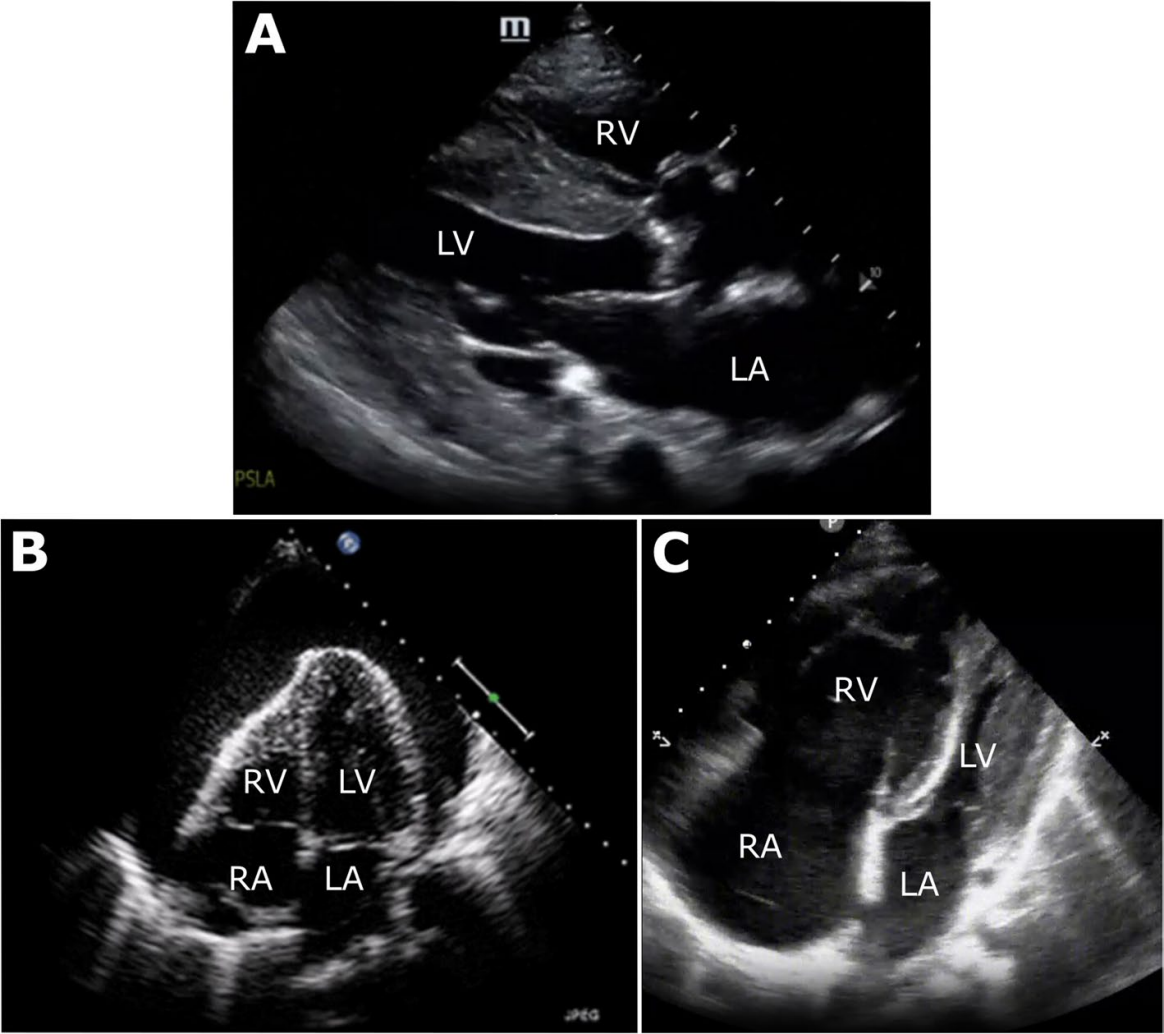
Focused cardiac ultrasound (FOCUS) findings which suggest congestive heart failure and volume overload. **A** Parasternal long-axis view demonstrating concentric left ventricular (LV) hypertrophy, which can be seen in heart failure with preserved ejection fraction. **B** A large pericardial effusion with evidence of right ventricular (RV) collapse, which may be seen in cardiac tamponade. Pericardial effusions can result from volume overload as well as numerous other etiologies. **C** Severe right atrial (RA) and RV enlargement, which can be seen in right-sided heart failure, typically resulting from pulmonary pathology. LA, left atrium

FOCUS consists of four cardiac views: parasternal long axis, parasternal short axis, apical, and subxiphoid [55]. FOCUS can evaluate cardiac pathology including pericardial effusion, valvular function, ventricular dysfunction, focal wall motion abnormalities, and chamber size (Fig. 3) [26]. Pericardial effusions, although most apparent on parasternal long-axis and subxiphoid views, can be visualized in any FOCUS view as a hypoechoic band adjacent to the heart, between the visceral and parietal pericardium (Fig. 3B) [26]. Although pericardial effusions can reflect the accumulation of fluid in decompensated CHF, there are numerous other potential etiologies, including infectious or inflammatory conditions, trauma, and renal failure, to name a few [56, 57].

The left ventricular (LV) systolic function can be assessed by FOCUS (Video S3, S4) [55, 58]. Features of normal systolic function include symmetrical inward LV wall movement in multiple views, fractional LV shortening > 25% by M-mode in the parasternal long-axis view, and symmetrical myocardial thickening; regional or global absence of any of these features can suggest systolic dysfunction [55, 58]. The LV ejection fraction can also be visually estimated by observing inward LV wall movement and reduction in chamber size between diastole and systole [55, 58].

LV chamber size and function can also be used to assess volume status in normal afterload states, where a large hypokinetic LV suggests volume overload, and a small hyperkinetic LV indicates volume depletion [26]. Caution must be taken, however, in over-attributing the LV cavity size to preload alone. For example, in low afterload states (e.g., sepsis), the cardiac function may appear hyperdynamic in the absence of hypovolemia. Conversely with high afterload states (e.g., severe hypertension, aortic stenosis), LV function may appear reduced in the absence of hypervolemia (Table 2) [59, 60].

Additionally, in conditions of elevated right ventricular (RV) pressures, the parasternal short-axis view can reveal a D-shaped LV due to the interventricular septum bowing towards the LV [61]. Specifically, a D-shaped LV in diastole only suggests volume overload, whereas a D-shaped LV in systole and diastole suggests pressure overload of the RV [61]. In a study of 526 patients undergoing echocardiography, both increased PA pressure and decreased RV function predicted the presence of a D-shaped LV [62].

Although right heart catheterization is the gold standard for diagnosis of pulmonary hypertension, transthoracic echocardiography and POCUS can also detect elevated PA pressures [63, 64]. Systolic PA pressure can be estimated using the maximum velocity of tricuspid regurgitation (TR) [63, 65]. First, the tricuspid valve is visualized in the four-chamber apical view, and continuous wave Doppler is applied to the TR jet to determine the maximum flow velocity [63, 65]. Then, the pressure difference between the RV and RA can be computed using the simplified Bernoulli equation, P = 4(TR_max_^2^), where P is the pressure difference and TR_max_ is the maximum velocity of TR [63]. During systole, the pulmonic valve is open, and PA pressure is equivalent to RV pressure. As a result, the systolic PA pressure estimate is the sum of the RAP and the difference between RAP and RV pressure, determined by the IVC assessment methodology [63, 65]. Given the potential for inaccuracy in IVC assessment, certain guidelines recommend assessing TR_max_ without incorporating RAP; TR_max_ < 2.8 m/sec suggests low probability, 2.9–3.4 m/sec intermediate probability, and > 3.4 m/sec high probability of pulmonary hypertension [63]. Additional findings that support the presence of pulmonary hypertension include RV hypertrophy or dilation, RA dilation, and a D-shaped left ventricle [63].

The TR-based method of assessing pulmonary hypertension is limited by several potential pitfalls (Table 2). TR_max_ may be underestimated in cases where the TR jet is poorly visualized due to limited sonographic windows [63]. Additionally, the presence of severe TR may also lead to underestimation of TR_max_ [63]. Beat-to-beat variability of TR_max_ in patients with atrial fibrillation or other arrhythmias may also limit accuracy, which can be mitigated by averaging measurements over several cardiac cycles.

Few data exist on serial POCUS PA pressure monitoring for volume status changes in patients with LV systolic dysfunction, but there is evidence that CHF therapy guided by invasive PA pressure monitors is effective at reducing hospitalizations [66]. Theoretically, POCUS could serve as a noninvasive alternative for serial PA pressure estimations to guide therapeutic intervention, though further studies are needed to investigate this possibility.

## Lung ultrasound

In addition to RA pressure assessments and FOCUS, lung POCUS can detect edema and congestion suggestive of volume overload in the setting of decompensated CHF [25].

### Pulmonary edema: B-lines

**Fig. 4:**
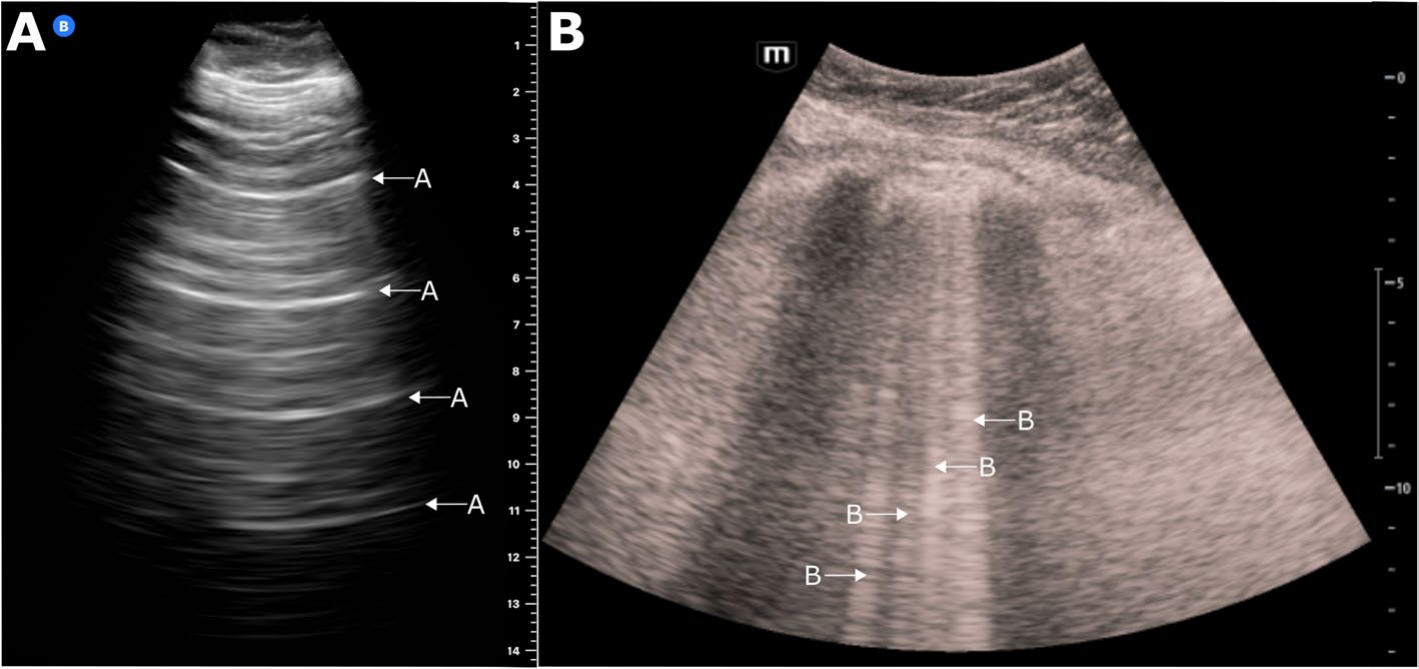
Common lung point-of-care ultrasound (POCUS) findings. **A** Normal lung POCUS without evidence of pulmonary edema. Arrows with the letter A identify A-lines, or normal artifacts representing reverberation of the pleura. **B** Lung POCUS indicating greater than 3 B-lines (identified by arrows with the letter B), which is diagnostic of pulmonary edema

The assessment of pulmonary edema by POCUS can be achieved with identification of B-lines [67]. B-lines occur when subpleural interlobular septa, which are normally subsonographic in resolution, become thickened and able to propagate ultrasound waves, thereby producing a hyperechoic reverberation artifact [67]. B-lines are hyperechoic ray- or comet-like reverberation artifacts which originate from the pleural line, move with lung sliding, and extend to the periphery of the far field (Fig. 4). Though numerous techniques have been described to evaluate B-lines, the bedside lung ultrasound in emergency (BLUE) protocol offers a standardized, efficient technique for detecting B-lines [68–70]. The BLUE protocol involves imaging six total points, comprised of three sites assessed bilaterally: the second intercostal space at the mid-clavicular line; the sixth or seventh intercostal space at the mid-axillary line; and the tenth to twelfth intercostal spaces at the posterior axillary line [68–70]. Although the BLUE protocol was originally described using a 5-MHz microconvex ultrasound probe, it can be replicated using curvilinear or phased-array probes [68–70]. Within this protocol, the presence of three or more B-lines at multiple, bilateral imaging points diagnosed pulmonary edema with 97% sensitivity and 95% specificity [68].

The distribution of B-lines also provides valuable information about their etiology; pathologic B-lines present in a diffuse, bilateral pattern can suggest cardiogenic pulmonary edema, whereas acute respiratory distress syndrome is more likely to produce a heterogenous pattern with some areas spared, and pulmonary fibrosis typically produces B-lines localized at the posterior lung bases [71].

A meta-analysis of lung ultrasound in acute decompensated heart failure reported that B-lines have a pooled sensitivity of 88% (95% CI, 75%–95%) and specificity of 90% (95% CI, 88%–92%) for detecting pulmonary edema, whereas chest x-ray has a sensitivity of 73% (95% CI, 70%–76%) and specificity of 90% (95% CI, 75%–97%) [72]. Studies included in the meta-analysis varied in ultrasound protocol, employing convex, curvilinear, or phased-array transducers at frequencies ranging from 2–5 MHz to assess two to six lung zones per hemi-thorax; ≥ 2 or ≥ 3 B-lines present in at least one lung zone bilaterally was considered positive for pulmonary edema [72]. Additionally, a greater number of B-lines on POCUS are associated with increased ventricular diameters, TR, and PA systolic pressures [73]. Prognostically, a study of 62 CHF patients showed that the presence of ≥ 19 total B-lines across eight imaging sites per patient was 57% sensitive and 86% specific for predicting in-hospital mortality [74]. It is important to note that B-lines do not uniquely reflect pulmonary edema from CHF and can be visualized in any condition that produces interlobular septal thickening, such as fluid from increased capillary permeability in infections, acute lung injury, or hemorrhage, or from deposition of fibrous tissue, collagen, or cellular debris from interstitial lung disease, sarcoidosis, or lymphangitic carcinomatosis, to name a few (Table 2) [67].

### Pleural effusions

In addition to B-lines, the presence of pleural effusions can suggest hypervolemia from CHF. Pleural effusions can be assessed sonographically by imaging the bilateral mid-axillary lines in the coronal plane at the level of the diaphragm. If present, an effusion appears as a hypoechoic space between the lung and diaphragm [75, 76]. POCUS is highly sensitive and specific for detecting pleural effusions, with sensitivity approaching 100% when > 100 mL of fluid are present [77]. A recent meta-analysis reported a pooled sensitivity of 95% (95% CI, 92%–97%) and specificity of 98% (95% CI, 96%–100%) for POCUS to detect pleural effusions, exceeding the accuracy of chest x-ray, which had a pooled sensitivity of 68% (95% CI, 58%–77%) and specificity of 85% (95% CI, 80%–91%) [77]. The finding of a pleural effusion is not specific for CHF, though, as many other conditions including pneumonia, malignancy, and volume overload due to non-cardiac conditions can produce effusions [78]. However, in the presence of known or suspected CHF, pleural effusions can indicate volume overload.

## Doppler assessment of venous congestion

**Fig. 5:**
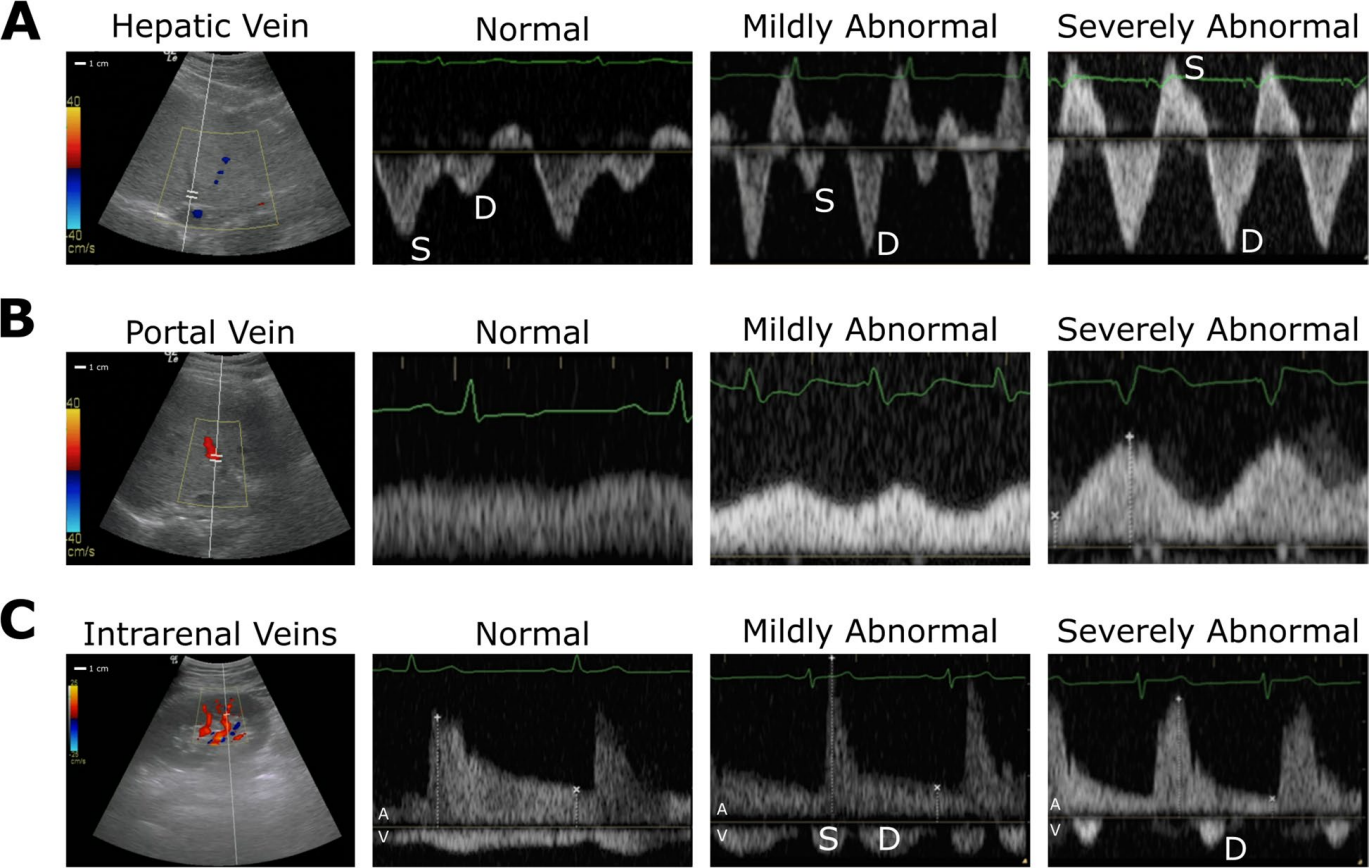
Representative hepatic, portal, and intrarenal vein imaging sites and pulse-wave Doppler signals obtained when performing a Venous Excess Ultrasound Score (VExUS). **A** Hepatic vein Doppler waveforms demonstrating normal (systolic [S] wave greater than diastolic [D] wave), mildly abnormal (D wave greater than S wave), and severely abnormal (S wave reversal) patterns. **B** Portal vein Doppler waveforms demonstrating normal (< 30% pulsatility index), mildly abnormal (30%–49% pulsatility index), and severely abnormal (≥ 50% pulsatility index) patterns; pulsatility index = (maximum velocity – minimum velocity)/maximum velocity. **C** Intrarenal vein Doppler arterial (A) and venous (V) waveforms demonstrating normal (monophasic flow), mildly abnormal (discontinuous, biphasic flow with systolic [S] and diastolic [D] phases), and severely abnormal (discontinuous, monophasic flow with diastolic [D] phase only). Reused from Dinh [82], with permission from POCUS 101

Doppler ultrasonography measurement of blood flow within the hepatic, portal, and renal veins throughout the cardiac cycle can further characterize volume status [25, 26, 79]. These values are integrated into the Venous Excess Ultrasound Score (VExUS) [79, 80]. In brief, the IVC is first assessed, and if it is less than 2 cm in diameter, congestion is ruled out [79]. If the IVC diameter is greater than 2 cm, then pulse-wave Doppler flow measurements in the portal, hepatic, and intrarenal veins are obtained [79]. Table 5 summarizes operational techniques used to obtain portal, hepatic, and intrarenal waveforms [79, 81]. Normal hepatic Doppler venous flow produces a biphasic pattern with a systolic (S) wave that is larger than the diastolic (D) wave (Fig. 5) [79, 82]. Flow is considered mildly abnormal if the D wave is larger than the S wave, and severely abnormal if the S wave is inverted (Fig. 5) [79, 82]. The portal vein Doppler produces a monophasic pattern, and a pulsatility index is computed by dividing the difference in maximum and minimum velocities by the maximum velocity; an index < 30% is considered normal, 30%–49% is considered mildly abnormal, and ≥ 50% is considered severely abnormal (Fig. 5) [79, 82]. Finally, normal intrarenal vein Doppler produces a monophasic pattern below the baseline, and is considered mildly abnormal when flow is discontinuous in a biphasic systolic and diastolic pattern, and severely abnormal when it is discontinuous with only diastolic flow (Fig. 5) [79, 82]. Overall, a VExUS assessment is graded on a 4-point scale, which is summarized in Table 6 [79].

**Table 5:** Operational characteristics for obtaining hepatic, portal, and intrarenal vein Doppler waveforms for the VExUS

VExUS site	Ultrasound probe	Imaging site	Doppler gate placement	Filter	Other tips
Hepatic veins	Curvilinear or phased array (2.5–5 MHz)	Right mid-axillary line at level of xyphoid process with indicator pointing toward axilla	Place sample 1–2 cm from hepatic vein and IVC junction	20–30 cm/sec	Expiratory hold without Valsalva; ECG gating
Portal veins	Curvilinear or phased array (2.5–5 MHz)	Right mid-axillary line at level of xyphoid process with indicator pointing toward axilla; caudal/anterior to hepatic vein	Place sample within distal main portal vein to avoid aliasing from hepatic artery	20–30 cm/sec	Expiratory hold without Valsalva; ECG gating
Intrarenal veins	Curvilinear or phased array (2.5–5 MHz)	Posterior axillary line at level of xyphoid process with indicator pointing toward axilla; posterior/caudal to hepatic vein	Align sample with interlobar vein	< 20 cm/sec	Decrease filter to find interlobar veins; ECG gating

ECG, electrocardiographic; IVC, inferior vena cava; VExUS, Venous Exess Ultrasound Score

**Table 6:** VExUS grading scale for severity of venous congestion

Grade	Significance	IVC diameter (cm)	No. of severely abnormal Doppler findings
0	No congestion	< 2	-
1	Mild congestion	> 2	0
2	Moderate congestion	> 2	1
3	Severe congestion	> 2	≥ 2

IVC, inferior vena cava; VExUS, Venous Exess Ultrasound Score

The VExUS score is associated with other markers of volume overload in CHF, including clinical assessment of CHF severity, N-terminal pro-BNP level, laboratory evidence of renal dysfunction, liver stiffness, and B-line count [83]. The VExUS score can also predict RAP more accurately than IVC measurements, with VExUS score of 3 predicting RAP ≥ 12 mmHg on right heart catheterization with 100% sensitivity and 85% specificity in one study [84]. In a prospective study of 74 patients with acute heart failure, a VExUS grade 3 assessment predicted death during hospitalization with a sensitivity of 80% and specificity of 75% [85]. The study also reported that acute heart failure readmission can be predicted at follow-up visit by an IVC above 2 cm (sensitivity, 93.1%; specificity, 58.3%) and a monophasic intrarenal pattern (sensitivity, 91.7%; specificity, 67.4%); additional VExUS measurements did not increase the accuracy of the prediction [85]. Notably, while VExUS is sensitive for venous congestion, it is not specific to CHF as the cause [79].

Altogether, current data suggest that VExUS is a useful tool to non-invasively obtain accurate assessments of venous congestion in heart failure patients. While VExUS grades 1 and 2 suggest the presence of congestion and can provide additional evidence that volume removal is appropriate, grade 3 findings are associated with significant congestion as well as mortality and should prompt a high degree of clinical concern [83–85]. There may be value in monitoring temporal changes in severity of individual venous waveforms (e.g., progression from a biphasic to monophasic intrarenal vein waveform, or progression from 45 to 55% portal vein Doppler pulsatility), though these changes would alter the overall VExUS grade in most cases [79, 85]. Because VExUS requires more time and operator experience than other POCUS techniques for volume assessment, we recommend reserving VExUS for cases when initial volume assessment (including IJV, IVC, and lung POCUS) is inconclusive or when specific clinical scenarios warrant a high degree of certainty in the volume status assessment. For example, VExUS could be used to confirm intrarenal venous congestion prior to diuretic escalation in a patient with suspected cardiorenal syndrome and worsening creatinine despite initial diuresis.

## Applying POCUS in the critically ill patient with heart failure

Critically ill patients with comorbid heart failure necessitate a nuanced approach to fluid management and resuscitation. For instance, a common clinical challenge is the management of sepsis in CHF patients. For this patient population, blindly administering the initial 30 mL/kg fluid bolus as recommended by the Surviving Sepsis Campaign may be detrimental [86]. POCUS can be invaluable in guiding the management of heart failure patients presenting with hypotension and shock, offering a tailored approach to assess fluid status and cardiac function.

A useful framework for evaluating patients with heart failure is the four classic hemodynamic profiles, first described by Forrester et al. [87] in 1976 (Table 7). A patient in profile 1 has adequate cardiac output (“warm”) without signs of organ congestion (“dry”) and is consequently characterized as "warm/dry." Profile 2 also has adequate cardiac output but is accompanied by congestion (“wet”), described as "warm/wet." Profile 3 is marked by reduced cardiac output (indicating hypoperfusion, “cold”) without congestion, termed "cold/dry." Lastly, profile 4 (“cold/wet”) presents with both low cardiac output and evidence of congestion. Each profile will have different goals for management [87].

**Table 7:** Classification of patients with congestive heart failure by cardiac output and volume status

Profile	Description	Management strategy
1 Warm/dry	Good cardiac output without congestion Vasodilated state with compensated fluid status	Patient will likely tolerate fluids. Pressors (norepinephrine) can be added counteract vasodilation
2 Warm/wet	Good cardiac output with congestion Vasodilated state but will have poor tolerance for additional fluid	Start pressors early and consider diuretics. Thoughtful administration of any additional fluid i.e., must show clear evidence of responsiveness/benefit
3 Cold/dry	Low cardiac output without congestion Common in early septic or hypovolemic shock. Volume would likely be helpful	Administer volume guided by serial POCUS assessment of fluid responsiveness and venous congestion
4 Cold/wet	Low cardiac output with congestion Improve cardiac function and fluid restrict	Consider inotropic therapy, diuretics, invasive cardiac output monitoring, and expert cardiology consultation

POCUS, point-of-care ultrasound

### Estimating cardiac output

The classification of “cold” versus “warm” designates a low vs adequate cardiac output state, respectively, in relation to organ perfusion. Findings including cold and clammy skin, mottled extremities, faint peripheral pulses, low pulse pressure, low urine output, rising blood lactate levels can all suggest a low cardiac output state, also known as cardiogenic shock. In addition to these clinical findings, right heart catheterization can measure cardiac output but requires an invasive procedure.

Stroke volume and cardiac output can also be estimated via cardiac POCUS by measuring the left ventricular outflow tract (LVOT) velocity time integral (VTI). This metric can be measured rapidly and non-invasively, making it a potential alternative to right heart catheterization. First, blood flow velocity through the LVOT is measured in an apical five-chamber view and the area under the flow velocity waveform is calculated to obtain the VTI. To ensure optimal measurement accuracy, the flow velocity waveform should be measured in parallel to the LVOT, and the VTI should be averaged over three to five beats (more in patients with beat-to-beat variability in cardiac output, such as those with atrial fibrillation). The LVOT diameter is also measured in parasternal long-axis view and can be used to estimate the CSA of the LVOT. The stroke volume is then calculated by multiplying the LVOT VTI by the CSA, and cardiac output is obtained by multiplying the stroke volume by the heart rate. This method provides a noninvasive estimate of the cardiac output [88]. Though LVOT VTI is a static measurement, it can be reassessed periodically to evaluate changes in cardiac output after interventions like fluid administration. A patient whose POCUS-derived cardiac output increases by > 15% following a fluid bolus is considered fluid responsive, as > 15% change exceeds the typical repeatability error of echocardiographic cardiac output measurements [89].

### Assessing fluid congestion

The classification into "wet" or "dry" reflects a patient's fluid status, particularly concerning organ congestion. Apart from traditional signs gathered from patient history, physical examinations, or elevated BNP levels, the POCUS findings discussed above like a distended IVC, VExUS scoring, presence of B-lines, and pleural effusions further substantiate the evaluation of venous congestion. A patient displaying these POCUS signs is not likely to tolerate additional fluid without exacerbating organ congestion.

### Impact of positive pressure ventilation

Many critically ill patients with CHF require positive pressure ventilation through noninvasive (continuous positive airway pressure, bilevel positive airway pressure) or invasive (endotracheal intubation) mechanisms, which can confound POCUS volume status assessment (Table 2). Positive pressure ventilation increases intrathoracic pressure during inhalation, decreasing venous return to the RA, which increases minimum IVC diameter and decreases IVC collapsibility [90, 91]. Likewise, as increasing positive end-expiratory pressure (PEEP) is applied to mechanically ventilated patients, preload decreases and afterload increases; this leads to an increased IVC diameter and decreased collapsibility [92]. In this context, the standard thresholds of IVC diameter > 2.1 cm and collapsibility < 50% do not reliably predict increased RAP [90, 93]. Current literature has not established adjusted measurement thresholds or imaging techniques to improve accuracy of RAP estimation in mechanically ventilated patients. Studies note the low diagnostic accuracy of using IVC changes in mechanically ventilated patients to predict fluid responsiveness compared to other transesophageal modalities [91], and there is large heterogeneity between studies [94], limiting its utility in mechanically ventilated patients. However, other studies demonstrate that in mechanically ventilated patients, end-expiratory IVC diameter < 8 mm predicts fluid responsiveness with 95% specificity, and IVC distensibility ratio ([maximum diameter – minimum diameter]/minimum diameter) > 18% predicts fluid responsiveness with 90% sensitivity and specificity, suggesting that identifying the presence of a small, collapsible IVC could remain useful for excluding volume overload in this population [90, 93, 95, 96]. Of note, IVC distensibility ratio is preferred to IVC-CI in mechanically ventilated patients [96].

Through the same mechanism, positive pressure ventilation increases IJV-CSA and IJV-CI, an effect which may be partially mitigated by assessing the right IJV with the patient’s head rotated 30 degrees to the left [97–99]. Like the IVC assessment, data suggest that IJV-CI remains useful for excluding volume overload, as an IJV-CI ≥ 11.4% predicts fluid responsiveness with 83% sensitivity and 94% specificity [97]. Additionally, B-lines in mechanically ventilated patients may reflect de-recruited segments of atelectatic lung, which can improve with increased PEEP, among other recruitment maneuvers [100]. In contrast, several studies have demonstrated no significant change in VExUS parameters with positive pressure ventilation [101–103].

Overall, IVC, IJV, and lung POCUS should be interpreted with caution in the context of mechanical ventilation, as positive pressure ventilation decreases the specificity of these exams for volume overload. IVC and IJV POCUS may still be useful as trends via serial exams for establishing fluid responsiveness, especially in the absence of changes in ventilator settings. In addition, VExUS should be routinely performed in mechanically ventilated patients, as it is less susceptible to confounding from positive pressure ventilation.

### A tailored approach to management

It is important to consider all available data, including physical signs and symptoms, laboratory results, and POCUS findings, to categorize a critically ill patient with heart failure into the appropriate cardiac output and volume status subgroup and dictate management strategy. In the intensive care unit (ICU) setting, the addition of POCUS to the evaluation of patients requiring mechanical ventilation was associated with decreased length of mechanical ventilation and ICU stay, as well as a lower fluid balance [104], suggesting that POCUS may increase the accuracy of volume assessment. Notably, patients’ volume status may fluctuate during their clinical course, so ongoing reassessment is critical. This stratified approach allows for personalized treatment plans and addresses specific needs indicated by their individualized hemodynamic profile.

## Additional considerations

### Special populations

In addition to the common pitfalls that can occur when using POCUS to assess volume status (Table 2), there are several populations that warrant special consideration with regard to the use of POCUS. One such population is patients with obesity. Although POCUS can detect signs of volume overload that may not be identified on physical examination in obese patients, such as JVD, increased subcutaneous fat and chest wall thickness may reduce image quality, limiting the diagnostic utility of ultrasound [25, 105]. Additionally, patients with chronic pulmonary hypertension and right heart failure commonly have findings of right heart pressure overload (D-shaped left ventricle) as well as signs of elevated CVP on IJV and IVC POCUS even in the absence of volume overload [106, 107]. Furthermore, additional caveats to POCUS interpretation are present in patients receiving advanced heart failure therapies, such as left ventricular assist device (LVAD) or heart transplant. Although LVADs generate continuous flow which alters arterial hemodynamics, sonographic assessments of cardiac function, CVP, and venous congestion can still be used [108]. However, views may be obscured by the device, and results must be interpreted in the context of current LVAD settings [108]. Additionally, changes in echocardiographic parameters including decreased RV function and dilation of the RA and RV commonly occur after heart transplant and improve over time; these findings do not necessarily reflect volume overload [109]. Generally, it is important to be aware that POCUS volume status assessment may be limited in patients with obesity, pulmonary hypertension, and advanced heart failure therapies.

### Training and competency

To accurately assess volume status with POCUS, a clinician must demonstrate competency in obtaining and interpreting relevant ultrasound images. Numerous POCUS training programs exist, and most include components of didactic education, instruction on image interpretation, and supervised image acquisition and interpretation [110]. The number of supervised exams required to become proficient varies based on the type of exam performed. For example, one study demonstrated 93% accuracy in lung ultrasound imaging and interpretation among physician and nonphysician trainees after 30 supervised exams, regardless of prior ultrasound experience [111]. The recommended number of supervised studies to achieve competency in lung ultrasound ranges from 25 to 50 based on guidelines from major emergency medicine, critical care medicine, echocardiography, and anesthesiology societies [110]. There is slightly more variation in the recommended number of supervised studies to become competent in FOCUS, with guidelines ranging from 25 to 150 [110]. Although fewer data are available on number of studies required to achieve competency with VExUS, one study demonstrated good interobserver reliability among clinicians who underwent a 4-h online training course, three in-person practice sessions, and performed 20 to 100 supervised exams [112]. Once proficiency is achieved, POCUS images used in medical decision-making should be saved within the electronic medical record for use in quality control and periodic review of competency [110]. Overall, we recommend that clinicians receive formal POCUS training before using this tool to augment their clinical volume status assessments.

## Proposed stepwise algorithm for POCUS volume status assessment

**Fig. 6:**
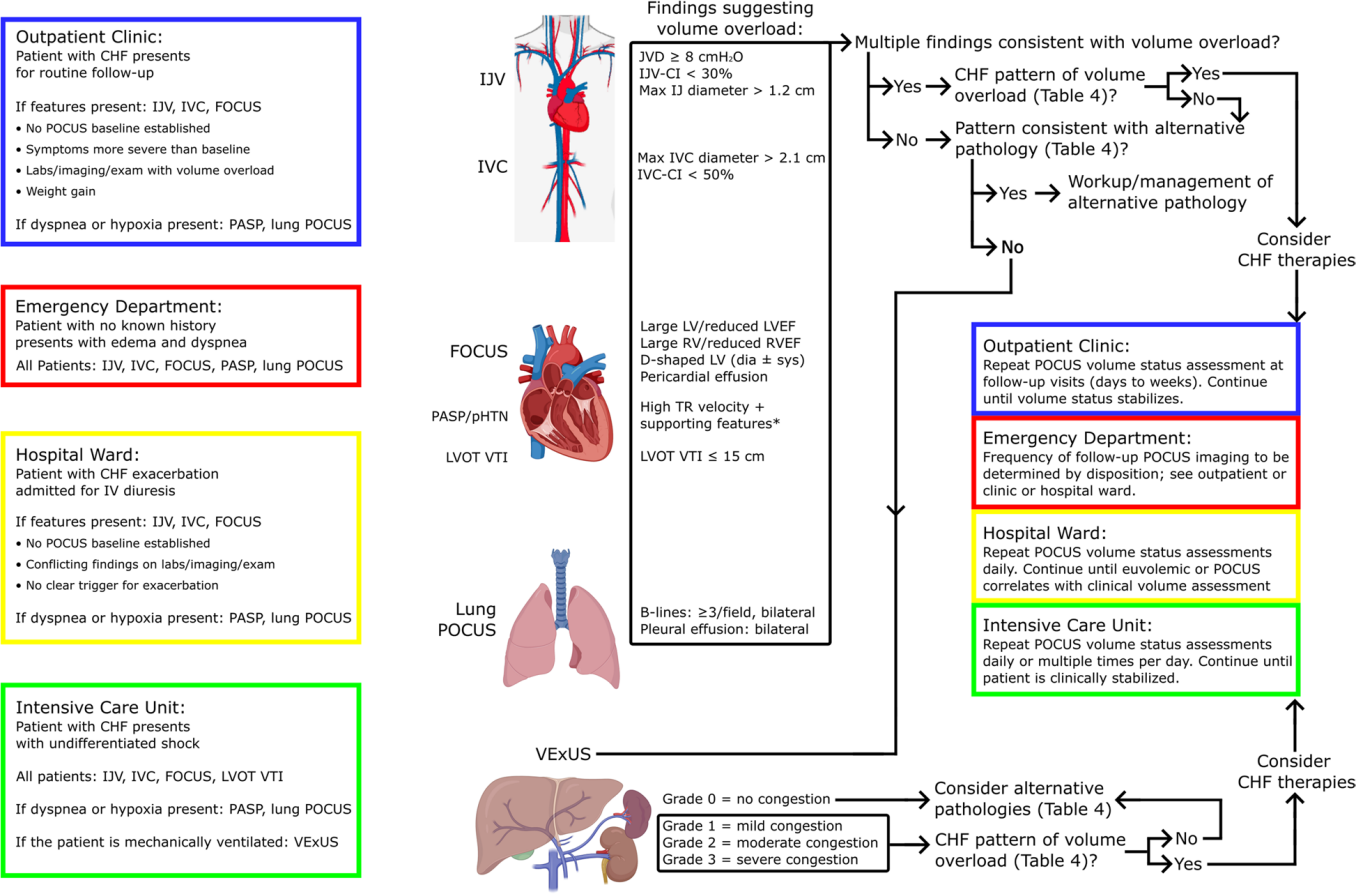
Proposed stepwise approach to point-of-care ultrasound (POCUS) for volume status assessment. Blue, red, yellow, and green boxes summarize POCUS studies that should be considered to evaluate the described clinical scenarios in outpatient, emergency department, inpatient ward, and intensive care settings, respectively. Black boxes and lines denote an approach to interpretation and further evaluation of the initial POCUS results. Proposed follow-up imaging intervals are listed in the blue, red, yellow, and green boxes on the right side of the diagram. This expert-opinion-based algorithm has not been prospectively validated; it should be used to supplement clinical judgement. *Supporting features for the presence of pulmonary hypertension include RV hypertrophy or dilation, RA dilation, and a D-shaped left ventricle. CHF, congestive heart failure; Dia, diastole; FOCUS, focused cardiac ultrasound; IJ, internal jugular; IJV, internal jugular vein; IJV-CI, internal jugular vein collapsibility index; IV, intravenous; IVC, inferior vena cava; IVC-CI, inferior vena cava collapsibility index; JVD, jugular venous distension; LV, left ventricle; LVEF, left ventricular ejection fraction; LVOT, left ventricular outflow tract; PASP, pulmonary artery systolic pressure; pHTN, pulmonary hypertension; POCUS, point-of-care ultrasound; RV, right ventricle; RVEF, right ventricular ejection fraction; Sys, systole; TR, tricuspid regurgitation; VExUS, Venous Excess Ultrasound Score; VTI, velocity time integral. Stock images obtained from BioRender [113]

Figure 6 presents a proposed stepwise approach to selection and interpretation of POCUS studies for volume status assessment in outpatient, emergency department, inpatient ward, and intensive care settings [113]. Importantly, this pathway is based on expert opinion and has not been prospectively validated. It is intended to supplement, not replace, clinical judgement. Briefly, we recommend the integration of POCUS in the volume assessment of any patient in the emergency or intensive care setting, while POCUS can be reserved for cases with atypical clinical features or unexpected symptom progression in outpatient clinics and inpatient wards. An initial assessment should include IJV, IVC, and FOCUS exams in all patients. Lung POCUS as well as evaluation for pulmonary hypertension via TR jet velocity and other supporting findings, such as RV hypertrophy or dilation, RA dilation, and a D-shaped left ventricle, should be performed in patients with dyspnea or hypoxia. VExUS should be performed in cases where volume status remains unclear after the initial ultrasound evaluation, and in all mechanically ventilated patients. If volume overload is established, serial examinations are key to monitoring response to treatment; frequency should vary by patient acuity, at least once daily in critically ill patients and less frequently in the outpatient setting.

## Conclusions

In patients with CHF, accurate assessments of volume status remain elusive. Alongside clinical symptoms, physical exam findings, imaging studies, and invasive monitoring, POCUS allows trained clinicians to glean information on a patient’s volume status with minimal cost or risk to the patient. Paired with traditional methods of volume assessment, POCUS has the potential to help trained clinicians better care for patients with CHF.

## Supplementary Information

Additional file 1: Video S1. The inferior vena cava is visualized deep to the liver; tracing its path cranially, towards the left side of the screen, reveals its junction with the right atrium.

Additional file 2: Video S2. Identification of the internal jugular vein (IJV) collapse point. The internal carotid artery can be seen deep to the IJV and its identity confirmed by its pulsatility. The identity of the IJV can also be confirmed by applying pressure to test for its collapsibility.

Additional file 3: Video S3. An apical four-chamber view demonstrating signs of congestive heart failure. The left ventricular ejection fraction is grossly reduced. Focal hypokinesis of the left ventricular anterolateral wall and apex suggests ischemic cardiomyopathy.

Additional file 4: Video S4. A parasternal short axis view demonstrating hypokinesis of the anterior and lateral walls suggestive of ischemic cardiomyopathy.

## Data Availability

No datasets were generated or analysed during the current study.

## Abbreviations

BLUE: Bedside lung ultrasound in emergency
BNP: Brain natriuretic peptide
CHF: Congestive heart failure
CI: Confidence interval
CSA: Cross-sectional area
CVP: Central venous pressure
FOCUS: Focused cardiac ultrasound
ICU: Intensive care unit
IJV: Internal jugular vein
IJV-CI: Internal jugular vein collapsibility index
IVC: Inferior vena cava
IVC-CI: Inferior vena cava collapsibility index
JVD: Jugular venous distension
LV: Left ventricle
LVAD: Left ventricular assist device
LVOT: Left ventricular outflow tract
PA: Pulmonary artery
PEEP: Positive end-expiratory pressure
POCUS: Point-of-care ultrasound
PPV: Positive predictive value
RA: Right atrium
RAP: Right atrial pressure
RV: Right ventricul
TR: Tricuspid regurgitation
VExUS: Venous Excess Ultrasound Score
VTI: Velocity time integral
